# Identification and Ranking of Core Values in Family Medicine: A Mixed Methods Study From Ukraine

**DOI:** 10.3389/fmed.2021.646276

**Published:** 2021-03-22

**Authors:** Pavlo Kolesnyk, Sabine Bayen, Ivanna Shushman, Andrew Kolesnyk, George Kuodza, Zalika Klemenc-Ketiš, Thomas Frese

**Affiliations:** ^1^Department of Family Medicine and Outpatient Care, Medical Faculty#2, Education Scientific Family Medicine Training Centre, Uzhgorod National University, Uzhgorod, Ukraine; ^2^Department of General Practice, University of Lille, Lille, France; ^3^Community Health Centre Ljubljana, Ljubljana, Slovenia; ^4^Department of Family Medicine, Faculty of Medicine, University of Maribor, Maribor, Slovenia; ^5^Department of Family Medicine, Faculty of Medicine, University of Ljubljana, Ljubljana, Slovenia; ^6^Department of General Practice & Family Medicine, Medical Faculty, Martin-Luther University Halle, Halle, Germany

**Keywords:** family medicine, core values, delphi, mixed methods, Ukraine

## Abstract

**Introduction/Context:** The term core value (CV) can be defined as fundamental beliefs or principles, guiding one's behavior in a social context. Though core competencies of family medicine (FM) have been clearly defined by WONCA, there has been an ongoing debate on what the CVs are for family doctors (FDs). Ukraine is a developing country in the middle of Europe with a population of 43 million inhabitants, gained independence from the Soviet Union in 1991. Ukraine is a low-income country, developing a modern European healthcare system, especially regarding FM. To implement WONCA standards, it is mandatory to assess the ongoing understanding of CVs in clinical daily practice among active FDs, working in different countries of Europe including Ukraine.

**Research questions:** How do Ukrainian FDs (Delphi group experts) define the CVs of FM in Ukraine and how important are these CVs to a wider population of Ukrainian FDs in their everyday practice?

**Methods:** A mixed method study was conducted in two steps during August and September 2020 in Ukraine. The first part was a qualitative Delphi round (three rounds) design among 20 Ukrainian FDs who were familiar with teaching and terms like CV. A consensus list of six CVs has emerged from the Delphi round study. The second part was a quantitative survey among Ukrainian FDs, who were not specially used to discussing CVs. The consensus list of those six CVs was then submitted to 2000 FDs (randomly selected) who were not involved in the Delphi team, to rank those values from one to nine, according to the importance from their personal point of view. Demographic characteristics have been assessed for all the participants of the Delphi round and quantitative survey.

**Results:** Twenty FDs were involved as experts in the first Delphi round, whereas only five experts continued their participation in the second and the third rounds of the survey. The following six CVs emerged from the Delphi round: comprehensive approach, care coordination, first recourse, continuity of care, integrated approach, and patient and family centered care. The final sample consisted of 375 FDs (19% response rate). There were 323 (88.7%) female and 34 (9.3%) male FDs in the sample. The mean age of the participants was 44.6±13.5 years.

**Discussion/Conclusion:** Defining CVs for FM by Ukrainian FDs in a given socio-economical and historical-cultural setting is crucial to optimize primary medical care and to guarantee an appropriate and successful implementation of WONCA standards as well as CVs in different countries including those where reformation of the health system is ongoing.

## Introduction

Ukraine is a low-income country with 44 million inhabitants in 2018 (1). In 2014, a reform of primary healthcare (PHC) was elaborated to replace the prior centralized model. Started in 2018, the reform is extended to secondary and tertiary levels in 2020 (2). According to the World Health Organization (WHO), providing healthcare (HC) for everyone with accent on PHC is the invariable key indicator of HC development (3). Based on the definition provided by WONCA, a Family Doctor (FD) is the first contact of all patients and provides comprehensive care to all individuals irrespective of age, sex, and illness (4). Following that WONCA definition, mandatory core competencies are expected to guide the major practices of FDs in most European countries including Ukraine. To provide this care effectively, the global association of FDs also listed core competencies which every specialist in FM should master: primary care management, person-centered care, specific problem-solving skills, comprehensive approach, community orientation, and holistic approach which are outlined schematically and known as the WONCA tree of competencies (5). The ministry of health accepted the WONCA competences for the Ukrainian health system concerning FM (6).

While the core competencies having been clearly defined by WONCA, the CVs have not been yet. There has been an ongoing debate on what are the CVs and core tasks for FDs (7–9). We will here focus on the definition of the CV, being defined as fundamental beliefs or principles which guide the behavior in a social context (10). Some authors consider CV as principles of the specialty that define the characteristics of FM (11). This means that CVs are crucial for both personal and professional development. Integrating values in practice is important because it provides room for both the patient-values and evidence-based medicine to be used together in decision making (12). Moreover, outlining the values necessary for an organization ensures that members of the team follow them. Defining CVs is crucial to guarantee optimal medical services, as observed in commercial businesses as well as in medical organizations (13). For other authors, including Ukrainian, the focus of CVs is more philosophical and theoretical and less related to clinical practice. One Ukrainian author divided values into two main groups', for example, humanistic and professional values. Humanistic values determine a person's behavior in society and his attitude to the world around him, while professional values determine a person's attitude to their professional responsibilities and regulate the professional activities of professionals (14). A first overview of 11 references related to CV and comprehensive care, documented since 1950, revealed that several values were commonly considered to be consensual (6–9).

These main CVs defined by different authors in FM were patient centered care, continuity of care, comprehensive care, and community-based care (11, 15–20). Furthermore, other values like generalist approach, reflective mindfulness, cost-effectiveness, care coordination, and continued medical education were mentioned. Some CVs may differ according to the clinical setting and available resources. For instance, community-based care is a well-recognized value among the majority of European countries as well as Ukraine, Canada, USA, Pakistan, New Zealand, and India. On the other hand, Sub-Saharan African references state that community-based care is not defined as a CV (6–9). It is known that these CVs have significantly changed over time and with the development of medicine and technology. Moreover, some of the CVs such as patient centeredness and continuity of care appear to be quite similar to the core competencies defined by WONCA. It is still unclear whether CVs can be core competences of FDs at the same time as long as they are interrelated and serve the global aim of FM. Our study aimed to define the CVs in FM from the Ukrainian FD's point of view, and to determine which of the defined CVs are the most important from them. Our research questions were:

- What are CVs in Family medicine from the Ukrainian FD's point of view?
- What is the level of importance given to each CV from the FD's point of view?

## Materials and Methods

### Study Design

A mixed method study was conducted during August and September 2020 in Ukraine. The first part of the study was a qualitative Delphi study among 20 Ukrainian FDs who were familiar with teaching and terms like CV, and the second part was a quantitative cross-sectional observational study among Ukrainian FDs.

Ethical approval – Approval was obtained by an Internal Review Board. The FDs (members of Ukrainian Family Medicine Association) contacted by e-mail were clearly informed about the study when they were invited to participate. The anonymization of their data especially was explained. They got a link to an anonymous online survey. The return of their completed questionnaires was considered as written consent.

### Part 1: Delphi Study to Determine the CVs of FM in Ukraine

We conducted three online rounds among 20 FDs (Table 1). We included active FDs involved in teaching, mentoring, and care, as far as these three dimensions represent Family Medicine as a university discipline. Thus, our Delphi experts had an academic and practical background at once, to get a comprehensive insight into the issue. Furthermore, we considered that each active FD, respecting good clinical practice, teaching, and mentoring the trainees as a trainer is an expert in FM. FD work in practice with patients and apply values and competencies in practice every day. Their opinion can be considered as an expert opinion and legitimize their participation in the Delphi round.

**Table 1:** Delphi-group participants' characteristics overview table with gender, age.

**Participant**	**Age**	**Gender**		**Working area**		**Practice**		**Level FM**		
	**Years**	**Male**	**Female**	**Urban**	**Rural**	**Group**	**Single**	**FDt**	**FDnt**	**T/R**
1st Round (*n* = 20)	33.5 ± 11.0	10%	90%	90%	10%	100%	0%	51%	34%	15%
2nd Round (*n* = 5)	29.8 ± 5.97	20%	80%	100%	0%	100%	0%	100%	0%	0%
3rd Round (*n* =5)	29.8 ± 5.97	20%	80%	100%	0%	100%	0%	100%	0%	0%

*FDt, family doctors who are involved in teaching*.

*FDnt, family doctors who are not involved in teaching*.

*T/R, trainees or residents*.

The group was moderated by one of Ukrainian researchers (PK) and assisted by the co-researchers (ZKK, SB, and TF).

The final result was a consensus list of six CVs. Furthermore, socio-demographic characteristics of participants were collected.

**Round 1:** The researchers asked the participants (*n* = 20) by e-mail to list CVs of family medicine according to their personal opinion. The authors gathered all the generated suggestions of all the participants to take out duplications, and to create a list of their CV.

**Round 2:** The researchers asked the participants to rate each CV from the list on a scale from 1 to 9: 1 meaning absolutely disagree that this is a CV, and 9 meaning absolutely agree that this is a CV. All CVs receiving <7 points on the Likert scale (*n* = 129) were excluded.

**Round 3**: The first author asked the participants to evaluate how much each of the listed items responds to the criteria of CV according to the scale from one to nine (where one does not respond and nine totally responds to the criteria of CV). Six items ranked from seven to nine were finally obtained.

### Part 2: Determining the Level of Importance of CVs

All the authors designed a simple cross-sectional study for FDs, members of the Ukrainian Association of FM from all regions (oblast) of Ukraine who did or did not have experience in mentoring trainees and also residents of FM. Among the 5,000 association members, 2,000 FDs were selected by systematic randomization and accepted an invitation to take part in the study by e-mail. The consensus list of CVs from Part 1 of the study was accessible by a link to an anonymous Google-form sheet by e-mail to the 2000 randomized Ukrainian FD. The participants were invited to rate each CV on a scale from one to four meaning one was not important at all and four was very important.

### Questionnaire

A standardized, anonymous, closed question questionnaire based on the consensus list of CV from Part one of the study was sent by e-mail to the 2000 randomized FDs. The following sociodemographic characteristics of participants were assessed: age, gender, urban/rural area of work, number of years of practicing as a FD, group/single practice, involvement in FD's training, being a trainee, and size of practice.

### Data Analysis

The data were collected in the Google form sheet and exported to an Excel sheet. The data analysis of the study Part two was performed, using the IBM SPSS Statistics 23. The following statistical tests were used: Cramer's V test, Chi-squared-test, and Spearman's rank correlation coefficient.

## Results

### Results of Study Part 1

#### Study Population

The 20 Delphi-round participants were all working FDs with different working experience. For the second and third round, the same five experts continued to follow the survey. They were all involved in teaching for at least 5 years.

#### Results of the Study Part 1 (Delphi Group)

The first round generated 56 CVs.

After removing the duplications of terms,49 CVs were left.

During the second round, these 49 CVs were ranked from one to nine.

**Figure 1:**
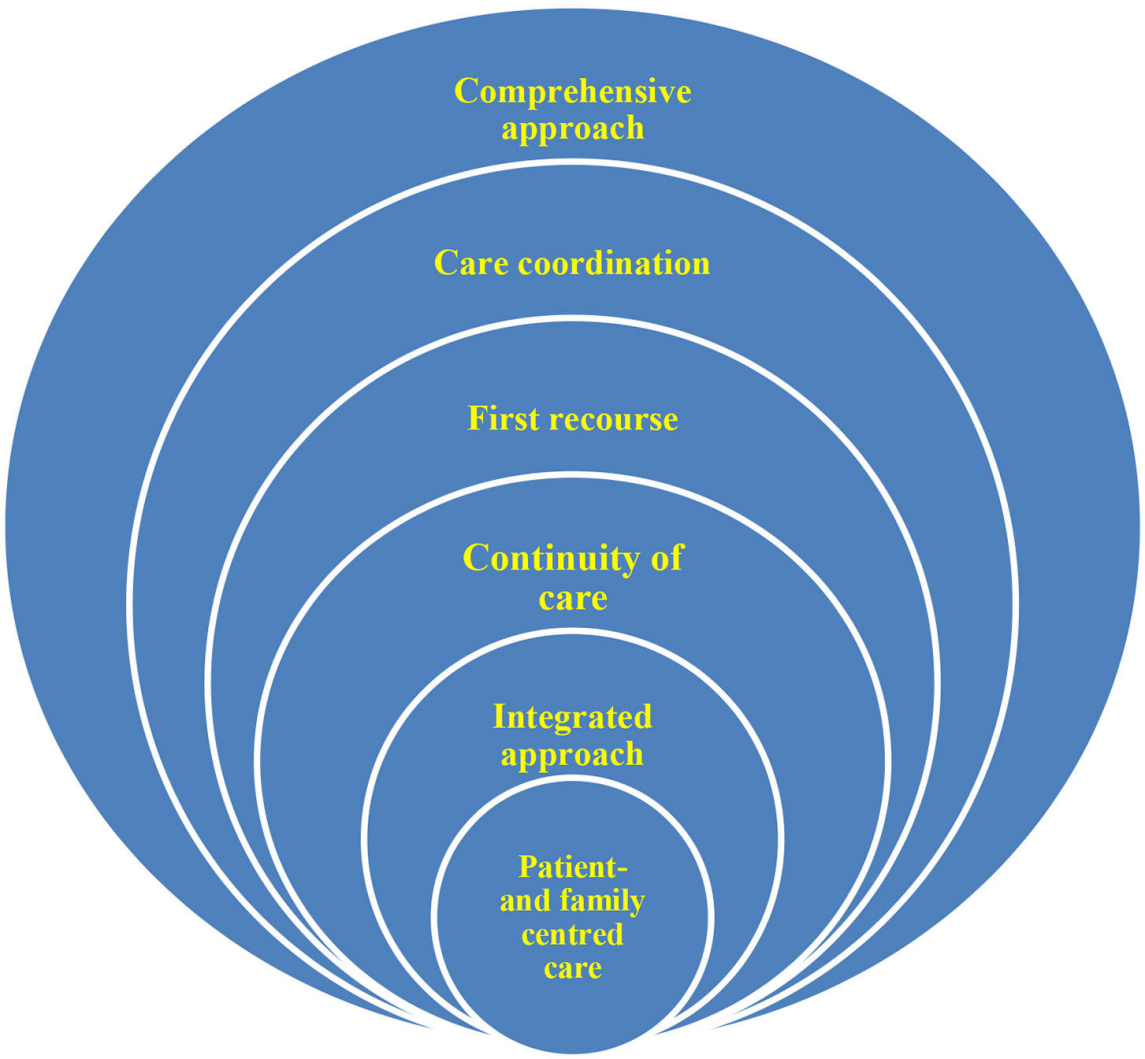
The six Ukrainian CVs.

As a result of the third round 6 consensual CVs had been finally generated out of the persistent CVs of the second round which accepted more than seven points on the scale from one to nine (Figure 1).

### The Results of Part Two of the Survey (Quantitative Online Survey)

#### Study Population

The final sample consisted of 375/2000 FDs and the response rate was 19% (Table 2).

**Table 2:** Characteristics of online survey participants.

**Participant**	**Age**	**Gender**		**Working area**		**Practice**		**Level FM**		
***n* = 375**	**Years**	**Male**	**Female**	**Urban**	**Rural**	**Group**	**Single**	**FDt**	**FDnt**	**T/R**
	44.6 ± 13.5	9.3%	88.7%	80.5%	19.5%	89.5%	10.5%	12%	79%	9%

*FDt, family doctors who are involved in teaching*.

*FDnt, family doctors who are not involved in teaching*.

*T/R, trainees or residents*.

There were 323 (88.7%) female and 34 (9.3%) male FDs in the selected group.

The mean age of the sample was 44.6±13.5 years.

#### The CV Ranking

**Figure 2:**
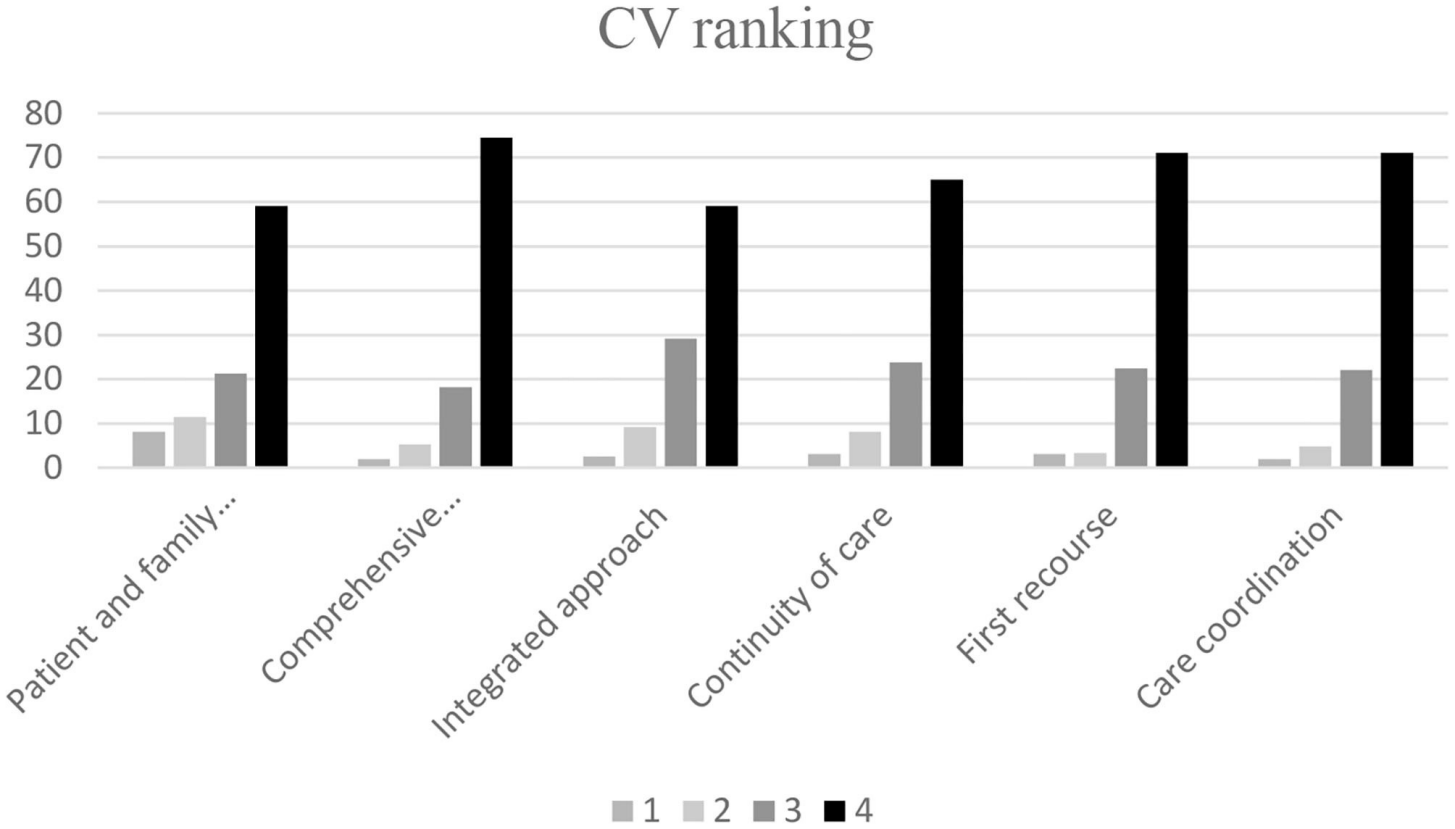
The CV ranking.

In the second part of the study, 375 FDs ranked the CVs from the list defined during the Delphi consensus (Figure 2 and Table 3). Table 4 shows the Spearman's rank correlations between the FD characteristics and their ranking of CVs.

**Table 3:** The CV ranking.

**Core value**	***n* (%) of 1 point ranking**	***n* (%) of 2 points ranking**	***n* (%) of 3 points ranking**	***n* (%) of four points ranking**
Care coordination	7 (2%)	17 (4.8%)	79 (22.1%)	254 (71.1%)
First recourse	11 (3.1%)	12 (3.4%)	80 (22.4%)	254 (71.1%)
Continuity of care	11 (3.1%)	29 (8.1%)	85 (23.8%)	232 (65.0%)
Integrated approach	9 (2.5%)	33 (9.2%)	104 (29.1%)	211 (59.1%)
Comprehensive approach	7 (2.0%)	19 (5.3%)	65 (18.2%)	266 (74.5%)
Patient and family centered care	29 (8.1%)	41 (11.5%)	76 (21.3%)	211 (59.1%)

*The KMO value was 0.813 and Bartlett's test was highly significant (p = 0.0001)*.

*Four significant low strength positive correlations (by Spearman's rank correlation coefficient) were found*.

**Table 4:** Spearman's rank correlations between the FD characteristics and their ranking of CVs.

**FD's characteristics**	**Core value**	**Rank correlation coefficient (*rs)***	***P*-value**
Age	Continuity of care	*0.117*	*0.028*
Term of work	First recourse	*0.143*	*0.007*
Term of work	Continuity of care	*0.178*	*0.001*
Term of work	Comprehensive approach	*0.138*	*0.01*

*Two significant low strength associations (by Cramer's V and Chi-squared tests) exist between teaching/training status (being involved/not involved in teaching/trainees or residents) and the CV “continuity of care”. As the older the FDs were, the more often they tended to rank the CV “continuity of care” as a very important CV*.

Table 5 shows the Cramer's V and Chi-squared associations between the FDs characteristics and their ranking of CV.

**Table 5:** Cramer's V and Chi-squared associations between the FDs characteristics and their ranking of CV.

**FD's characteristics**	**Core value**	**1 = not important at all**	**2 = not important**	**3 = important**	**4 = very important**	**Cramar's V, Chi-squared tests statistics**
FD not being involved in teaching FD being involved in teaching	Continuity of care Continuity of care	3% 2.2%	7.4 % 13%	21% 19.6%	68.6% 65.2%	V = 0.155, *p* = 0.009χ^2^ = 16.961,
Trainees/residents	Continuity of care	5.7%	5.7%	48.6%	40%	*p* = 0.009
Men	Care coordination	5.8%	5.8%	41.2%	52.9%	V = 0.157, *p* = 0.035
Women	Care coordination	6.6%	6.6%	19.8%	73.6%	χ^2^ = 8.623, *p* = 0.035

*“Continuity of care” as a CV, was ranked as very important by all the FDs, regardless of their teaching experience. The majority of the trainees ranked the core value of “continuity of care” as important or very important. The majority of male FDs ranked “care coordination” as very important or important. The majority of female FDs ranked “care coordination” as very important*.

## Discussion

This was the first nationwide study about CVs among 375 FDs in Ukraine. The vast majority of respondents were female FDs at an average age of 44 years, which is representative of the national average gender/age of FDs (11).

Six CVs emerged and were ranked by the participants, according to their perceived importance.

The CVs identified in the Ukrainian context correspond to previously identified CVs in several documents over the last years. For example, continuity of care, comprehensive care, care coordination, and complex care have been identified in 1991 by the Society of Teachers of FM in the USA, the Royal New Zealand College of GPs, and in Pakistan (12).

The enrolled Ukrainian FDs determined their CVs as deeply held views that act as guiding beliefs for individuals and organizations. CVs state clearly what the professional stands for, and they should remain the same through time (13).

This is an important finding, given the specific sociocultural background which Ukraine represents compared to some other European countries, especially regarding the ongoing implementation of the primary care system (PCS). This could indicate that CVs of FM are universal regardless of the different countries' features.

During the last few years, FM became more popular in Ukraine because according to the last legislation FDs became main paid doctors even comparing with the secondary and tertiary care levels (11).

Ukraine is on the way to develop a PCS according to the WHO standards. High quality service has to follow some appropriate requirements: to be comprehensive, accessible, coordinated and integrated, and to ensure continuity. All characteristics outlined in the WHO framework have to be considered equally by all health systems to improve the overall health system (14).

The comprehensive approach was ranked as one of the most important CVs for Ukrainian FDs. Comprehensive approach is listed in the European Definition of FM as one of the core competencies.

According to Starfield (15), a comprehensive or well-developed primary care system has the following characteristics: access to care, continuity of care, comprehensiveness, and care coordination. Ukrainian respondents who took part in the survey defined those characteristics as CVs.

### Care Coordination and “Gate Keepers”

Being the patient's first recourse and care coordination, especially patient orientation, were equally considered to be the second most important CV. The majority of male FDs ranked “care coordination” as very important or important. The majority of female FD ranked “care coordination” as very important. This result is comprehensive as far as, despite the ongoing transformation of the PCS, geographical disparities in access to care and therefore care coordination, still exist in 2020 (11).

According to the Regional Office for Europe of the World Health Organization in 2009 (11), the new GP-based system is much more prevalent in rural areas (with little access to specialized care), than in urban areas. At present, newly trained FDs do not always work according to the intended primary care model. It is not yet common for FDs to provide medical care to all age groups. Children are still often treated by pediatricians. Though the official job description allows FDs to cover general internal medicine, pediatrics, obstetrics/gynecology, family planning and reproductive health, tuberculosis, HIV/AIDS, health education and sanitary–epidemiological services (11).

FDs do not often provide their whole spectrum of services consistently throughout the country. Their tasks are poorly defined, the required knowledge and skills are not well-delineated, hindering requirements for recertification, and the flat salary without any incentive to provide the full range of services, are current obstacles to deploy FM all over the country (11).

Consequently, patients are aware of this situation and do not require many FD services. The FDs' working conditions, especially in rural areas, do not foster the services they are supposed to provide to their patients (11).

The Ukrainian healthcare system recognizes the importance of FD but still suffers from a global lack of FDs in the state. The number of doctors per capita in Ukraine is 47 doctors per 10,000 population, which is twice as high as in Western Europe. In large cities, such as Kiev, Donetsk and Dnipropetrovsk, this number was even higher: 80 doctors per 10 000 population, but only 3% of doctors were FDs only a decade ago. Now their number has become higher and is coming up to 7% (16).

Ukraine has 19 medical schools certifying over 10,000 graduates annually (2), and has 3.0 doctors per 1,000 population (1).

Only 17% of physicians in Ukraine are working at primary care level. Of these, one third are physicians who have been retrained as GPs. Using the official norms for calculation, the current number of retrained GPs/FDs amounts to 22% of the number required to cover the whole population with general practice/family medicine-based primary care (17).

However, in recent years the PCS based on FM has been successfully reorganized in Ukraine, with payment according to capitation, increased salaries for FDs—general practitioners and an national electronic management system (6, 18). In 2019 25.000 out of 186.178 Ukrainian doctors were officially registered in the e-system of medical health as FDs (19).

Many successful steps including financial efforts have already been made to provide Ukrainian citizens “full access to state-guaranteed medical services.” Through an agreement of the National Health Service of Ukraine, all establishments are expected to be autonomous and provided with the help of computerized net, so “gate-keeping” and coordination of the patient's care system has become a reality in Ukraine in the last few years (20).

Integrated approach and comprehensive care as terms were later recognized in the 1990s but the idea of comprehensive care was present before in Ukrainian literature. However, they have been earlier defined by Ukrainian authors as tasks but not as a CV (6). The “integrated approach” and “patient and family centered care” were equally ranked by FDs participants of the survey.

Continuity of care, which has been defined as a CV by the Scandinavian colleagues, is closely related to “patient centered care” as it helps to build a mutual trust and a better doctor-patient relationship to provide high quality care for the patient (21). It is mentioned as a provision of care for the patient from the prenatal stage to the geriatric age that enriches the physician's knowledge and experience, as well as after bringing the patient back to the primary medical care from the other levels of medical care. This principle was also well presented in Ukrainian literature before as a competence, but not as a CV (6). “Continuity of care” was ranked less among the six important CVs. The older the FDs were, the more often they tended to rank the CV “continuity of care” as a very important CV. This result is not surprising as far as FM is characterized by a biopsychosocial long-term follow-up (4). It is interesting to note that trainees also tended to rank the CV “continuity of care” as a very important CV. We can suppose that teaching of FM today includes the defined CV.

Medical education in Ukraine includes only 1 to 2 weeks of Family Medicine in the sixth year compared to 4–8 weeks in other European countries. Perhaps the lack of earlier exposure to FM can explain the lack of FDs in Ukraine. Sometimes, a lack of exposure to academic role models, and lack of primary care-based research has been accused of avoiding FM.

FD training involves a two-year residency (6), with 10 months of “theoretical training” (lectures, seminars, case discussions) and a year of “practical training” in family medicine clinics (17). Practical FDs who are usually not trainers and do not often have any academic experience take part in supervising residency training during their practical year of residency.

### Patient-and Family Centered Care

We could consider person-centered care as a CV, task, and competence at once, as far as it is a central and universal element of FM (22–26). It is a CV to aim person-centered communication to guarantee the respect and trust. It is a task to use the appropriate language to allow the patient's comprehension of diagnosis and care projects. It is a competence to be able to master different ways and skills of communication and to choose the most appropriate among them for a person met in a given moment of his life, presenting an individual situation and care demand. In Ukrainian literature and legislation this definition is outlined as a competence but not as a CV or a task and was not clearly stated (6).

Some of the identified CVs (not only in Ukraine but also in other countries) are listed as core competencies in the European Definition of Family Medicine (4). It seems that it is still not totally clear what CV means and what it is aimed for. This issue should be addressed in Ukrainian and European contexts to be able to identify the real CVs of FM.

## Strengths and Limitations

We conducted the first nationwide study to explore the perception and importance of CVs from the Ukrainian FD's perspective. That is original, new, true, and useful. Our mixed method study design was original and appropriate to approach such an abstract topic as CV. We first explored the topic through a qualitative approach to define concrete CVs. Then, we invited 2,000 FD, less familiar with theoretical CVs, to give us their opinion about the importance of each suggested CV. This is a democratic, bottom-up approach, likely to promote CVs among the concerned population of FDs, all members of the Ukrainian Association of Family Medicine (UAFM), which was founded in 1998. The UAFM became a WONCA member in 1999, and territorially entered the European region. UAFM is an all-Ukrainian public organization that links not only FDs and nurses, but all those who support the ideas and share the philosophy of FM. UAFM currently has more than 10,000 members, who are also members of regional (oblast) associations and branches. The main goal of UACM is to improve the health of Ukrainians by improving the quality of medical care to the population of Ukraine on the basis of family medicine.

Only five experts out of the 20 Delphi experts agreed to participate in the second round. It was surprising, but we still succeeded to motivate them to continue the Delphi round until the end.

Actually, as far as it was the first time that Ukrainian FD took part in such qualitative study, they were very enthusiastic in the first round, but then declined to follow the next two rounds, not used to this type of time-consuming participation. Regarding the response rate of the second quantitative part of the study, we obtained 19%, which seems low, but it is known that surveys through e-mail contact generally show lower response rates than web-based surveys (27). Maybe there was also a lack of interest and time from the FD's side because of the COVID-19 pandemic workload (28).

According to the Regional Office for Europe of the World Health Organization FD recruitment, retention of staff and poor motivation may be related to low prestige of medical workers in primary care. Potential solutions to promote and improve working conditions in FM are provision of information, publicity to the public, and strengthening of the position of FM in the academic world (11).

Nevertheless, our results, despite this lower response rate, probably derived from a smaller group of progressive physicians, are still important to report. Our mixed method study design could be easily applied in primary care settings of other countries, regardless of their stage of evolution of their primary care system.

## Conclusion

For the first time in history of Ukraine, CVs of FM were identified and ranked according to their importance by Ukrainian FDs. The identified CVs of FM globally corresponded to those previously defined by the WHO framework, in different countries in the world.

This finding indicates that CVs of FM are similar on the international level, regardless of the cultural, organizational, societal, and geographical features of a country. Identifying CVs of FM in different socio-cultural settings is important to harmonize and expend good clinical and ethical practice according to the WONCA framework of core competencies and core values. Especially evolving Primary Care systems could benefit from this guidance to guarantee equal access to FM in a world of increasing mobility of patients and FDs. It could be useful to teach these newly defined CVs to the next generation of Ukrainian FDs to cultivate them in future practice.

## Data Availability Statement

The raw data supporting the conclusions of this article will be made available by the authors, without undue reservation.

## Author Contributions

PK supervised and coordinated the study. SB, PK, TF, and ZK-K designed the study, supervised the data collection process, and developed the draft through their discussions. GK and IS did the literature review. AK did the data analysis. SB did the first draft. All authors contributed to the article and approved the submitted version.

## Conflict of Interest

The authors declare that the research was conducted in the absence of any commercial or financial relationships that could be construed as a potential conflict of interest.
