# Supplementary material for: Identification and Ranking of Core Values in Family Medicine: A Mixed Methods Study From Ukraine
Source: Front Med (Lausanne). 2021 Mar 22;8:646276. doi: 10.3389/fmed.2021.646276 (PMC8019795; doi:10.3389/fmed.2021.646276)
Supplement: Supplementary file 1 [file Data_Sheet_1.pdf]

1 Supplementary Material

The quantitative questionnaire

Introduction:

To offer optimal and equal care to patients all over Europe and worldwide, WONCA is going to list the ***CV*** *for Family Doctors . I.e we need opinion of FDs in different countries to clarify them. At present, no clear consensus has been found* ***to classify CV***

We are interested in your personal point of view concerning your comprehension of the list of CV in family medicine in Ukraine.

The general definition (not medical) of values and tasks (Oxford Languages):

Value: *Principles or standards of behavior; one's judgement of what is important in life.*

We would like to clarify your subjective understanding of what CV in FM in Ukraine you can define :

According to your point of view what the CV of FM in Ukraine: (at least 5)

1

2

3

4

5

6

7

8

9

10

Demographical data

Anonymous

age,

gender,

urban/rural area of work

N years of practicing as a FD,

group/single practice.

Teacher (or better: are you involved in training?)

Are you trainee

Size of practice
